# Study on the influence of meteorological factors on influenza in different regions and predictions based on an LSTM algorithm

**DOI:** 10.1186/s12889-022-14299-y

**Published:** 2022-12-13

**Authors:** Hansong Zhu, Si Chen, Wen Lu, Kaizhi Chen, Yulin Feng, Zhonghang Xie, Zhifang Zhang, Lingfang Li, Jianming Ou, Guangmin Chen

**Affiliations:** 1 Emergency Response and Epidemic Management Institute, Fujian Center for Disease Control and Prevention, Fuzhou, 350012 Fujian China; 2 Fujian Provincial Key Laboratory of Zoonosis Research, Fuzhou, 350012 Fujian China; 3 The practice base on the school of public health Fujian Medical University, Fuzhou, 350012 Fujian China; 4 Climate Assessment Office of Fujian Climate Center, Fuzhou, 350007 Fujian China; 5 Shengli Clinical Medical College of Fujian Medical University, Department of Health Management of Fujian Provincial Hospital, Fuzhou, 350001 Fujian China; 6 College of Computer and Data Science, Fuzhou University, Fuzhou, 350108 Fujian China; 7 School of Public Health, Fujian Medical University, Fujian 350108 Fuzhou, China; 8 Science and Technology Information and Management, Fujian Center for Disease Control and Prevention, Fuzhou, 350012 Fujian China

**Keywords:** Meteorological, Influenza, DLNM, LSTM

## Abstract

**Background:**

Influenza epidemics pose a threat to human health. It has been reported that meteorological factors (MFs) are associated with influenza. This study aimed to explore the similarities and differences between the influences of more comprehensive MFs on influenza in cities with different economic, geographical and climatic characteristics in Fujian Province. Then, the information was used to predict the daily number of cases of influenza in various cities based on MFs to provide bases for early warning systems and outbreak prevention.

**Method:**

Distributed lag nonlinear models (DLNMs) were used to analyse the influence of MFs on influenza in different regions of Fujian Province from 2010 to 2021. Long short-term memory (LSTM) was used to train and model daily cases of influenza in 2010–2018, 2010–2019, and 2010–2020 based on meteorological daily values. Daily cases of influenza in 2019, 2020 and 2021 were predicted. The root mean squared error (RMSE), mean absolute error (MAE), mean absolute percentage error (MAPE) and symmetric mean absolute percentage error (SMAPE) were used to quantify the accuracy of model predictions.

**Results:**

The cumulative effect of low and high values of air pressure (PRS), air temperature (TEM), air temperature difference (TEMD) and sunshine duration (SSD) on the risk of influenza was obvious. Low (< 979 hPa), medium (983 to 987 hPa) and high (> 112 hPa) PRS were associated with a higher risk of influenza in women, children aged 0 to 12 years, and rural populations. Low (< 9 °C) and high (> 23 °C) TEM were risk factors for influenza in four cities. Wind speed (WIN) had a more significant effect on the risk of influenza in the ≥ 60-year-old group. Low (< 40%) and high (> 80%) relative humidity (RHU) in Fuzhou and Xiamen had a significant effect on influenza. When PRS was between 1005–1015 hPa, RHU > 60%, PRE was low, TEM was between 10–20 °C, and WIN was low, the interaction between different MFs and influenza was most obvious. The RMSE, MAE, MAPE, and SMAPE evaluation indices of the predictions in 2019, 2020 and 2021 were low, and the prediction accuracy was high.

**Conclusion:**

All eight MFs studied had an impact on influenza in four cities, but there were similarities and differences. The LSTM model, combined with these eight MFs, was highly accurate in predicting the daily cases of influenza. These MFs and prediction models could be incorporated into the influenza early warning and prediction system of each city and used as a reference to formulate prevention strategies for relevant departments.

**Supplementary Information:**

The online version contains supplementary material available at 10.1186/s12889-022-14299-y.

## Background

Influenza is an acute infectious respiratory disease caused by influenza viruses [1]. Globally, there are an estimated 1 billion cases, 3 million to 5 million severe cases, and 290,000 to 650,000 influenza-related respiratory deaths each year, according to data from 1999 to 2015 [2]. Until late 2017, the World Health Organization (WHO) estimated that seasonal influenza was associated with a total of 250,000 to 500,000 deaths from all causes annually [3, 4]. In early 2019, a publication from the Global Burden of Disease Study (GBD) estimated a range of 99,000 to 200,000 annual deaths from lower respiratory tract infections directly attributable to influenza [3, 5]. When an influenza pandemic occurs, this number increases dramatically [6]. Using preliminary data obtained during the influenza season, the US Centers for Disease Control and Prevention (CDC) estimated that there were between 32.0 and 43.4 million influenza illnesses, between 401,000 and 706,000 hospitalizations, and between 27,300 and 49,000 influenza-associated deaths during 2018–2019 [7, 8]. The number of influenza reports in China increased substantially from 2018 to 2019, and the incidence rate increased from 55.09/100,000 in 2018 to 253.36/100,000 in 2019 [9]. Among them, the proportion of influenza-like illness (ILI) in the total number of outpatient and emergency cases (ILI%) in the southern region reached 8%, and the positive rate of nucleic acid detection of ILI samples was nearly 50%. The epidemic trend of influenza in China dropped precipitously in the early stage of the coronavirus disease 2019 (COVID-19) epidemic, reaching a very low level in history due to the COVID-19 lockdown effect. However, the intensity of the influenza epidemic in China has gradually increased since the autumn and winter of 2021. In particular, considering that the severe COVID-19 pandemic continues, COVID-19 and other respiratory infectious diseases may overlap [10]. Influenza is highly contagious, the speed of transmission is fast, the population is generally susceptible, it is easy to trigger a cluster of epidemics in schools and kindergartens, the social impact is large, and the burden of disease is serious.

**Fig. 1:**
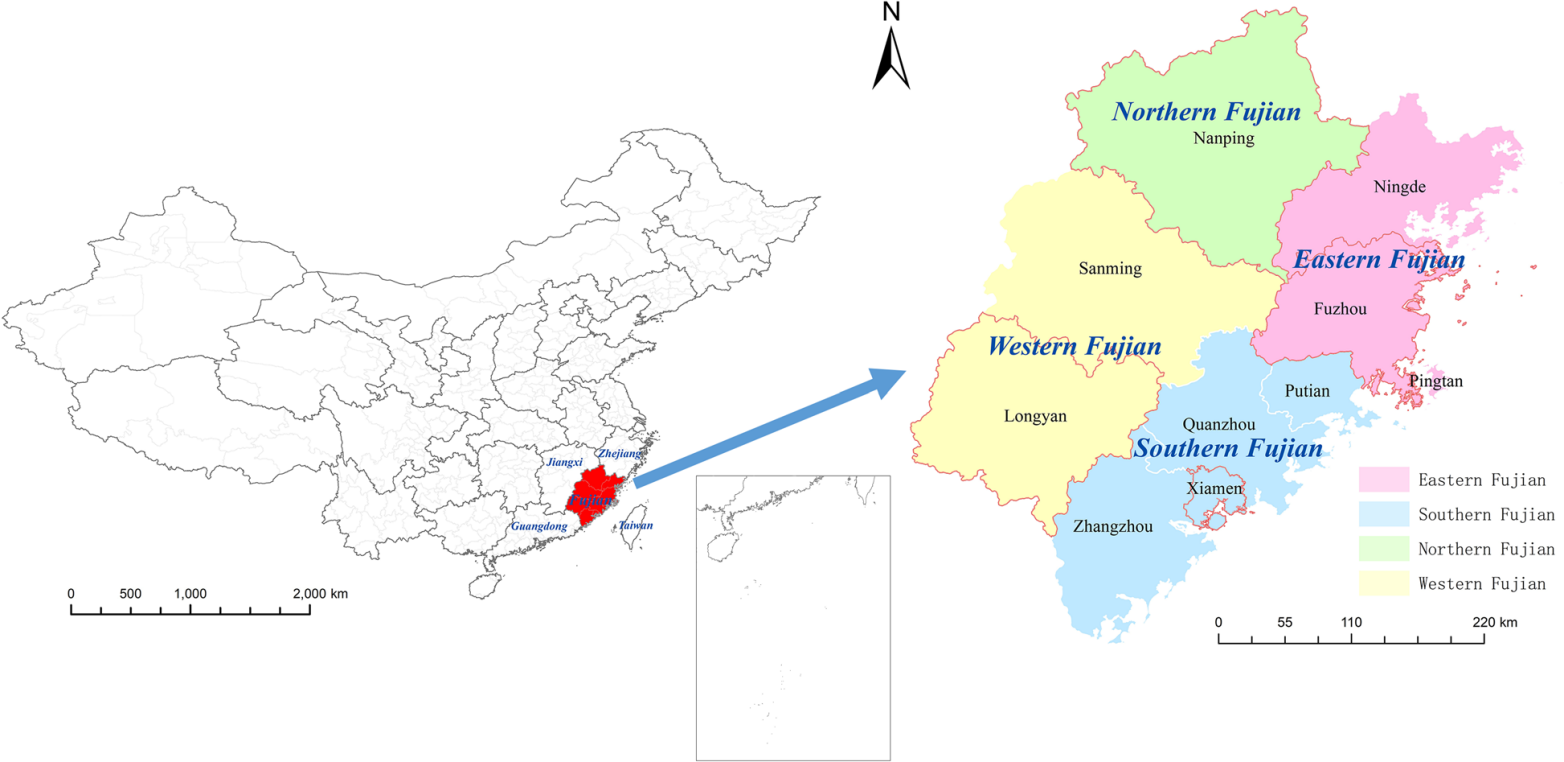
Location of Fujian Province in China and regional distribution. (Note: The red box indicates the location of the four cities analysed in this study: Fuzhou, Xiamen, Nanping and Longyan)

Fujian Province, abbreviated as "Min", is a provincial administrative region of the People's Republic of China located on the southeast coast of China (Fig. 1), with a total land area of 124,000 square kilometres and a geographical location between 23°33' and 28°20' north latitude and 115°50' and 120°40' east longitude. Fujian Province has a subtropical oceanic monsoon climate that is warm and humid. However, the climate in the region varies greatly; the coastal areas of southeast Fujian belong to the tropical climate zone of South Asia and northeast Fujian, northern Fujian and western Fujian are in the central subtropical climate zone. The vertical differentiation of hydrothermal conditions in each climate zone is apparent (Supplemental Fig. 1).

Many studies have shown an association between MFs and influenza, and temperature, sunshine, humidity and precipitation are usually used for research. Research reports have shown that low temperature, low humidity and less sunshine can increase the transmission of influenza [11–16]. However, it has been reported that both low and high temperatures can increase the intensity of influenza activity [16, 17]. Different research results showed that the average relative humidity was not significantly correlated with influenza incidence, but there was a significant positive correlation between average relative humidity at lag 6 and the incidence of influenza, indicating that the relative humidity also had a positively correlated long-term lag effect on influenza incidence [15]. In contrast to Gomez-Barroso et al.'s research results showing a statistically significant increase in influenza transmission in relation to an increase in the quantity of precipitation, Soebiyanto et al. reported that influenza associations with precipitation were location-dependent and inconclusive [13, 14]. Moreover, Wang et al.'s study indicated that high air pressure increased the risk of influenza [12]. Data types (e.g., daily data, weekly data), analysis model schemes, and region-specific characteristics (e.g., the spatial distribution of the weather stations, socioeconomic factors, terrain, living environment, etc.) may have led to these differences in research reports [18]. For instance, the use of weekly averages for meteorological parameters and unreasonable meteorological information collection stations could have affected the level of precision [13].

There are 9 cities and 1 comprehensive experimental development zone in Fujian Province, and the marked meteorological regional heterogeneity is substantial. Thus, it is not suitable to use a single meteorological and influenza association model for assessing and predicting influenza in the province.

Therefore, this study focused on four representative cities in different regions of Fujian Province (eastern Fujian, southern Fujian, northern Fujian and western Fujian) (Fig. 1) and analysed the MFs that are not often considered: wind speed (WIN), air pressure difference (PRSD), and air temperature difference (TEMD). Fuzhou and Xiamen are coastal cities, with air pressure and wind speed increasing from inland to coastal areas and precipitation decreasing. Fuzhou is the capital of Fujian Province and the political, economic and cultural centre of the province. Xiamen is densely populated and economically developed and is a special economic zone of China, with high temperatures and more sunshine hours. Nanping and Longyan both belong to inland mountainous areas, and the wind speed is low. Nanping is located at a higher latitude and the average annual temperature and minimum temperature are low; the average annual pressure in Longyan is low (Supplemental Fig. 1).

In this study, distributed lag nonlinear models (DLNMs) were proposed to analyse the characteristics, similarities and differences of meteorological influences on influenza in four cities to provide a basis for predicting influenza through meteorology.

Influenza cases were then predicted in the four cities. The latest research report confirms that in the field of time series data analysis and prediction with complex relationships (such as disease real-time analysis and prediction), compared with traditional machine learning methods, long short-term memory (LSTM) in deep learning models yields better results [19–24]. In this study, LSTM combined with MFs was proposed to predict the daily cases of influenza in the four cities to provide technical support for the construction of an influenza prediction and early warning system in each city and help relevant departments formulate prevention strategies.

## Materials and methods

### Data sources

The influenza case and population data of in Fujian Province from January 1, 2010, to December 31, 2021, were derived from the China Disease Prevention and Control Information System. The China Disease Prevention and Control Information System is a monitoring and reporting system for infectious diseases, chronic diseases and injuries established by the Chinese government in 2003. At present, it covers all medical and health institutions at and above the township health centre level in Fujian Province. While realizing real-time case reporting, the system also realizes dynamic and rapid statistical analysis of monitoring data and early monitoring of disease outbreak information. Influenza cases were defined as laboratory-confirmed cases and cases clinically diagnosed by medical and health institutions at all levels according to the diagnostic criteria for epidemic disease cases (WS 285–2008) [25], excluding suspected cases: (A) clinically diagnosed cases included an epidemiological history and acute high fever (axillary temperature ≥ 38 °C), chills, headache, aches and pains or other symptoms and signs; (B) confirmed cases included clinically diagnosed and ILI cases with positive laboratory test results of influenza samples. Among them, ILI refers to fever (axillary temperature ≥ 38 °C), accompanied by cough or sore throat, and a lack of other laboratory diagnostic basis. In Supplemental Fig. 1, the meteorological means of Fujian Province from 1991 to 2020 were derived from the Fujian Climate Center. The daily meteorological value data of Fujian Province from January 1, 2010, to December 31, 2021, were derived from the meteorological data network of the China Meteorological Administration (http://data.cma.cn). Then, the missing values were proofread and supplemented by the Fujian Climate Center on the state 30-year average climate daily data in China surface climate standard value data derived from this website. The MFs in this study included 8 daily value indicators: air pressure (PRS, hPa), PRSD (hPa), relative humidity (RHU, %), precipitation (PRE, mm), air temperature (TEM, °C), TEMD (°C), WIN (m/s), and sunshine duration (SSD, h). PRS, RHU, TEM and WIN were measured as daily averages, PRE was measured as the daily cumulative precipitation, SSD was measured as the number of sunshine hours, PRSD was defined as the difference between the maximum and minimum values of daily air pressure, and TEMD was defined as the difference between the highest and lowest values of daily temperature. The number of lag days in this study referred to the number of days delayed by the date of influenza onset compared to the statistical date of the corresponding MFs.

### Statistical analysis of data

Regional maps in Fig. 1 and Supplemental Fig. 1 were drawn using ArcGIS (version 10.2, ESRI, Redlands, CA, USA). In Supplemental Fig. 1, kriging interpolation was used for drawing [26, 27].

R software (version 4.1.0, R Foundation for Statistical Computing, Vienna, Austria) was used to statistically analyse the daily cases of influenza and daily meteorological value data, in which the population with influenza was stratified by sex (male and female), age (0 ~ 12 years, 13 ~ 59 years, ≥ 60 years) and area (urban and rural), of which the age-stratified group was divided according to the epidemiological characteristics of influenza in Fujian Province. First, a simple analysis of influenza and MFs was conducted, and the number of influenza cases per day in the four cities was summarized. Then, the differences in daily sequence values between sexes and regions were tested by t test, and the differences in daily sequence values between age groups were tested by F test. Differences in daily sequence values between the total number of influenza cases and MF values among the four cities were examined by F test. Differences with *P* < 0.05 were considered statistically significant. Then, the time series for the variables were plotted. The Pearson correlation analysis plot between MFs and influenza was generated. Finally, a DLNM was used to analyse the influence of MFs on influenza. The interaction diagrams between different MFs and influenza were plotted.

The DLNM incorporates both nonlinear dependency and delay effects, with the essential goal of adding a lag dimension to the exposure–response relationship through a cross-basis function, thereby describing the variation distribution of its effects in both the independent and lagging dimensions [28]. A cross-base matrix for meteorological data and the daily incidence of influenza was established, and the quasi-Poisson connection function was used for estimation. After controlling for the effects of day of the week, seasonality and long-term trend [29, 30], the relationship between meteorological factors and influenza was fitted using the DLNM model. The basic model is as follows:

1 $$log\left[E(Yt)\right]\mathit\;=\alpha\mathit\;\mathit+\beta ixi\mathit\;\mathit+\mathit\;NS\mathit\;\left(Zj,\;df\right)\mathit\;\mathit+\mathit\;Dow$$

*Yt* is the t-day number of influenza cases, *α* is the constant term, *xi* is the influencing factor, *βi* is the coefficient, *Zj* is the potential confounding factor, *Dow* is the dummy variable for the effect of the day of the week, *df* is the degree of freedom, and *NS (…)* is a natural spline function. *Df* was determined by the Akaike information criterion (AIC) minimum criterion, which ultimately determined that PRS, PRSD, RHU, PRE, TEM, TEMD, WIN, and SSD were all defined as 3. Accounting for the incubation period, epidemic characteristics and pretest results of influenza, the maximum number of lag days was determined to be 14 days, and lag days that had cumulative effects of MFs on the risk of influenza in each population were 3 d, 7 d and 14 d. The average of each MF was used as a reference value. The meteorological grade values were set according to the RR value of the pretest, which was used to analyse the cumulative effect on influenza in each population stratification. The MFs of the pretest included the minimum value, the median value, the average value, the maximum value and other values. Meteorological values with higher or more typical RR values were adopted. The grading value of MFs is shown in Table 1.

**Table 1:** Grading values of MFs used to analyse the cumulative effect on influenza in each population stratification

**Variables**	**Fuzhou**				**Xiamen**				**Nanping**				**Longyan**			
	**1**	**2**	**3**	**4**	**1**	**2**	**3**	**4**	**1**	**2**	**3**	**4**	**1**	**2**	**3**	**4**
PRS (hPa)	979	994	1012	1026	975	988	1008	1017	968	985	1000	1015	950	965	975	990
PRSD (hPa)	2	8	16	24	2	12	20	39	2	4	8	15	2	4	8	12
RHU (%)	27	60	80	100	23	60	80	100	36	60	75	100	25	60	85	100
PRE (mm)	0	3	30	244	0	4	30	172	0	3	60	154	0	3	60	152
TEM (°C)	3	10	23	33	4	13	23	32	-1	4	13	31	2	13	23	31
TEMD (°C)	1	5	10	17	1	5	10	17	1	5	10	21	2	6	13	22
WIN (m/s)	1	3	6	9	1	4	6	9	2	3	—	—	1	3	5	7
SSD (h)	0	2	8	12	0	2	8	12	0	2	8	12	0	2	8	12

Python (version 3.8.13, Python Software Foundation, Delaware, USA) and Tensorflow (version 2.8.0, Google Brain Team, Mountain View, CA, USA) were used to predict the daily cases of influenza through LSTM combined with MFs.

LSTM is an artificial intelligence deep learning algorithm suitable for time series data analysis. Its key feature is the ability to connect the network model in front of and behind neurons so that the network can process the time series data from both directions. The basic principle of LSTM is shown in Supplemental Attachment 1.

The root mean square error (RMSE), mean absolute error (MAE), mean absolute percentage error (MAPE) and symmetric mean absolute percentage error (SMAPE) were used to quantify the accuracy of the model's predictions, and the smaller the value was, the higher the prediction accuracy and the higher the confidence (best value = 0; worst value =  + ∞) [21, 31, 32].

The RMSE calculation formula is as follows:

2 $$\mathrm{RMSE}=\sqrt{\frac{1}{\mathrm{n}}{\sum }_{i=1}^{n}{\left({P}_{i}-{X}_{i}\right)}^{2}}$$

The MAE calculation formula is as follows:

3 $$\mathrm{MAE}=\frac1n{\textstyle\sum_{i=1}^n}\mid P_i-X_i\mid$$

The MAPE calculation formula is as follows:

4 $$\mathrm{MAPE}=\frac{100\%}n{\textstyle\sum_{i=1}^n}\mid\frac{P_i-X_i}{P_i}\mid$$

The SMAPE calculation formula is as follows:

5 $$\mathrm{SMAPE}=\frac{100\%}n{\textstyle\sum_{i=1}^n}\frac{\mid P_i-X_i\mid}{\mid\frac{\mid P_i\mid+\mid X_i\mid}2\mid}$$

In the above formulas, *Pi* is the observed daily incidence of influenza cases on the *i* day, and *Xi* is the predicted daily incidence of influenza cases on the *i* day where *i* = 1…, *n*.

## Results

### Descriptive statistics

In total, 136,199 influenza cases were reported over the study period in Fujian Province, with an incidence rate of 353.70/100,000 people. Fuzhou, Xiamen, Nanping and Longyan reported 13,729 cases, 21,324 cases, 4,315 cases and 5,696 cases, and the incidence rates (1/100,000) were 191.22, 563.83, 160.83 and 217.19, respectively. There were significant differences between sex, age, and urban and rural groups for influenza in the four cities (*P* < 0.001). The total influenza cases and various MFs varied significantly among the four cities (*P* < 0.001). The detailed characteristics of influenza and MFs are presented in Table 2 and Supplemental Table 1.

**Table 2:** Stratified influenza characteristics of populations in the four cities based on daily cases

**Variables**	**Sex**		**Age (years)**			**Area**	
	**Males**	**Females**	**0 ~ 12**	**13 ~ 59**	**60 ~ **	**Urban**	**Rural**
Total cases	25,092	19,972	23,624	19,375	2,065	27,133	17,931
Constituent ratio(%)	55.68	44.32	52.42	42.99	4.58	60.21	39.79
t/F	36.44		259.40			33.76	
p	0.00		0.00			0.00	

**Fig. 2:**
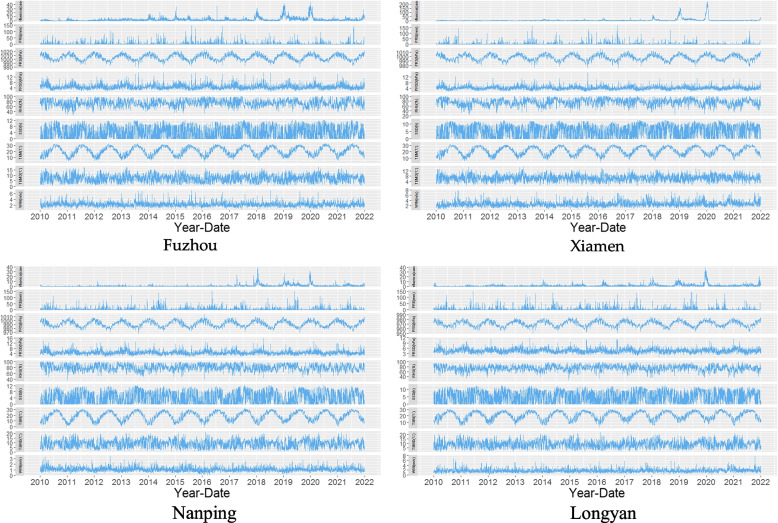
Time series of influenza cases and MFs in the four cities. (Note: Some extremely high MF values were not included in these maps)

Figure 2 shows the time series of influenza cases and MFs in the four cities, revealing that there was a certain seasonal periodicity in the daily values of influenza, PRS, PRSD, RHU, PRE, TEM, TEMD, WIN and SSD in the four cities, and there was a certain consistency in their fluctuations. This indicates that there may be a correlation and lag between influenza and MFs.

### Correlation analysis

**Fig. 3:**
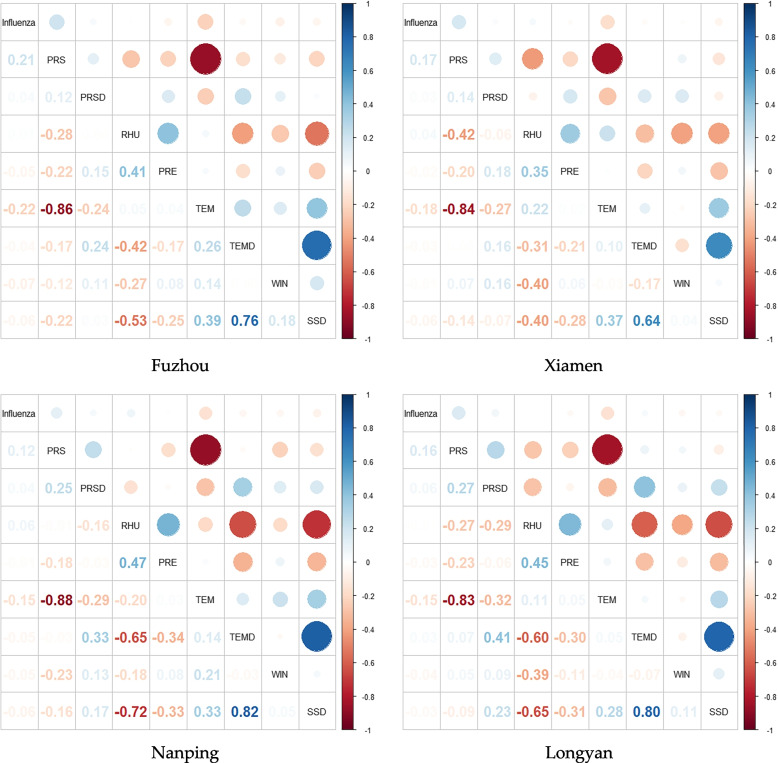
Pearson correlation analysis of influenza and MFs

There were highly positive and negative correlations between influenza and PRS and TEM in all four cities, among which the correlation values (r) in Fuzhou were 0.21 and 0.22, respectively. Between most of the MFs in the four cities, the correlations between PRS and TEM and between SSD and TEMD were strongest. However, there were inconsistencies among the four cities. For instance, there was a positive correlation between RHU and TEM in Xiamen, Longyan and Fuzhou, among which Xiamen had a stronger correlation (*r* = 0.22); however, Nanping had a negative correlation (*r* = -0.20). There was an obvious positive correlation between PRSD and SSD in Nanping and Longyan, while there was a negative correlation in Xiamen, but the correlation between them was not obvious in Fuzhou. The detailed correlations between influenza and various MFs are presented in Fig. 3.

### DLNM analysis

The cumulative effect a PRS (< 985 hPa) lag of 6 to 8 days showed risk factors, and the peak was at 7 cumulative days. However, there was a difference between the low PRS peaks in the four cities, with the peaks in Fuzhou and Xiamen concentrated at 975–979 hPa and Longyan appearing at 964 hPa (RR: 1.09, 95% CI: 1.01–1.46). An extreme PRS lag of 0 to 2 days was a risk factor for influenza (Longyan, 950 hPa, lag 1 d, RR = 1.56, 95% CI: 0.78–3.10). The cumulative effect of a high pressure (> 112 hPa) lag of 7 to 14 days on the risk of influenza gradually increased, but the peak of high pressure in Longyan was only 990 hPa (lag 14 d, RR: 6.01, 95% CI: 1.05–34.30).

High PRSD (> 8 hPa) had a significant effect on influenza and increased rapidly with increasing PRSD, and the cumulative risk effect increased with increasing lag time and then gradually decreased, reaching a peak with a lag of 7 days (Nanping, 15 hPa, RR = 30.04, 95% CI: 0.58–1544.04).

The cumulative effect of low RHU (< 40%) on the risk of influenza in Fuzhou and Xiamen was obvious, while high values (> 80%) in Fuzhou and Longyan were obvious (Fuzhou, 100%, lag 7 d, RR = 1.86, 95% CI: 1.17–2.97).

The cumulative effect of PRE on the risk of influenza in Xiamen and Longyan increased with the increase in PRE, among which PRE in Xiamen gradually decreased with the increase in the number of lag days (172 mm, lag 0 d, RR = 3.52, 95% CI: 1.11–11.20), whereas it first decreased and then increased in Longyan.

Low (< 9 °C) and high (> 23 °C) TEM were risk factors for influenza in the four cities, but there were also differences. TEMs in Fuzhou and Xiamen at 10 to 15 °C were also risk factors (Fuzhou, 10 °C, lag 14 d, RR = 1.84, 95% CI: 1.18–2.88), showing a wave type between 3 and 33 °C, while Nanping and Longyan exhibited a "U" shape between -2 and 32 °C. The cumulative effect of low temperature on the risk of influenza first increased and then decreased with the increase in the number of lag days, while high temperature risk first decreased and then increased.

Both low (< 3 °C) and high (> 8 °C) TEMDs were risk factors for influenza. Low TEMD had cumulative effects on the risk of influenza in Fuzhou, Xiamen and Nanping, and the cumulative effect of high TEMD risk increased with increasing TEMD (Nanping, 21 °C, lag 7 d, RR = 3.72, 95% CI: 1.06–13.08). However, the cumulative effect of influenza risk in Fuzhou and Xiamen weakened rapidly at a TEMD > 12 °C.

With the increase in WIN (> 6 m/s, Nanping was > 2 m/s), the risk of influenza gradually increased; WIN in Fuzhou and Longyan first decreased and then increased with the increase in lag time, whereas WIN in Xiamen and Nanping continued to increase with the increase in lag time.

SSD was a risk factor for influenza at 1 to 4 h and 11 to 13 h. The cumulative effect of high SSD on the risk of influenza decreased with increasing lag time and then increased. The cumulative effect of low SSD risk first decreased and then increased with increasing lag time. Nanping was mainly at greater risk of influenza development when SSD was small (0 h, lag 14 d, RR = 3.88, 95% CI: 1.08–14.01).

**Fig. 4:**
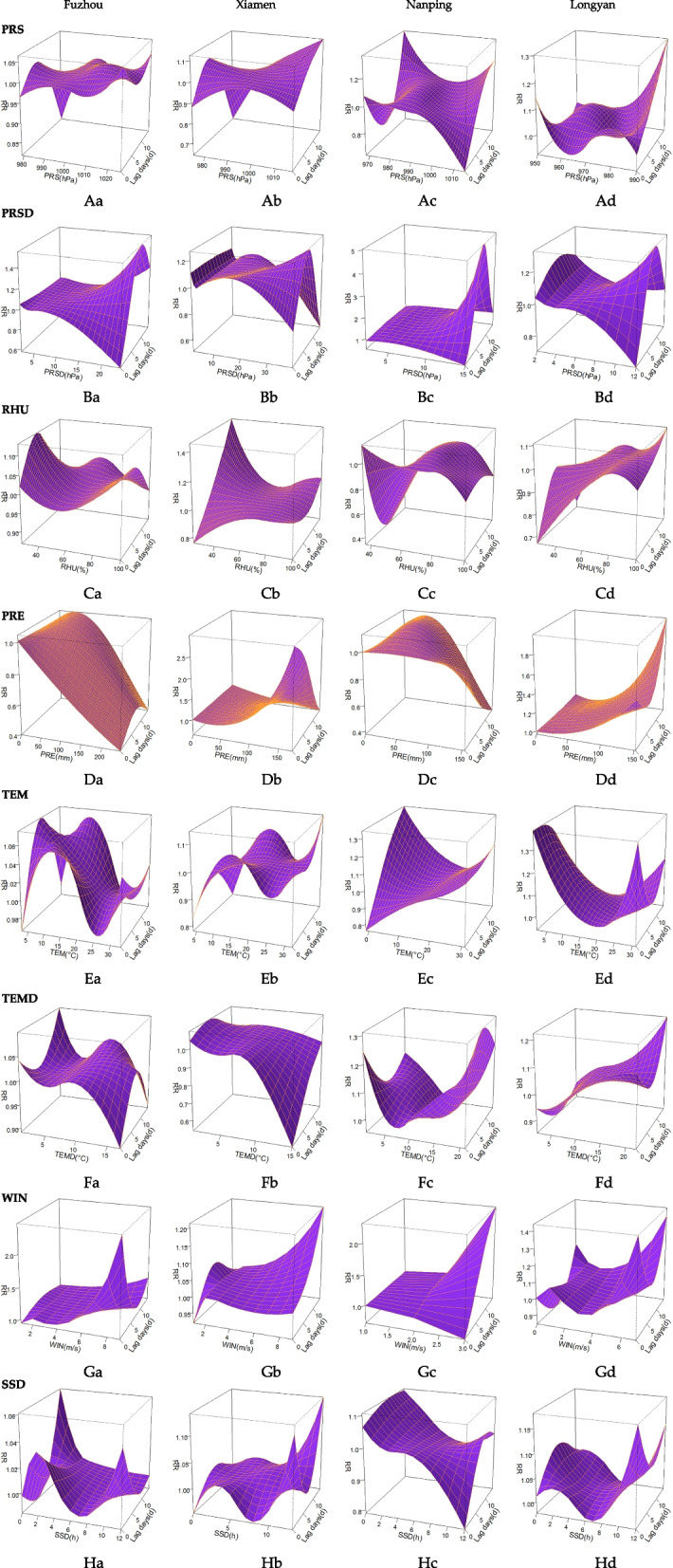
3D plots of the effects of MFs on influenza

Additional characteristics of the impact of MFs on influenza in the four cities are presented in Fig. 4.

### Population stratification analysis

Low (< 979 hPa), medium (983–987 hPa), and high (> 112 hPa) PRS were risk factors for women, children aged 0–12 years, and rural populations, with the cumulative risk effect of high PRS being more significant and increasing with the increase in lag time (the low, medium and high PRS values in Longyan were < 955 hPa, 972–978 hPa, and > 980 hPa, respectively).

The cumulative effect of influenza risk among women increased with increased PRSD and lag days. Low PRSD (< 8 hPa) was more significant in the ≥ 60-year-old population and increased with increasing lag time (Longyan, 4 Pa, lag 14 d, RR = 1.97, 95% CI: 0.80–4.86). Compared with urban areas in the four cities, considering the coverage of PRSD values that had a significant positive correlation with influenza, lag time span and RR values, the risk of PRSD on influenza was more significant in rural areas.

The cumulative effect of RHU on influenza among women was significant when the lag time was long (≥ 7 d). Compared with men, the effect of high PRE on influenza among women in Xiamen, Nanping and Longyan was more significant.

The cumulative effect of both low and high TEMs on the risk of influenza among women was more significant. The cumulative effect of low TEM on the risk of influenza in the 0 ~ 12-year-old group and ≥ 60-year-old group in Xiamen was more significant.

The effect of high WIN on influenza in the ≥ 60-year-old population was more significant and increased with the increase in WIN and lag days. However, in Nanping, the effect of WIN on influenza was more obvious in the 0 ~ 12-year-old group (3 m/s, lag 3 d, RR = 3.46, 95% CI: 0.50–23.88). Compared with urban areas, WIN had a more significant impact on influenza in rural populations.

The cumulative effect of high SSD (> 7 h) on influenza among women people ≥ 60 years old and urban populations was more significant. The effect of low SSD on influenza in rural populations was more significant.

**Fig. 5:**
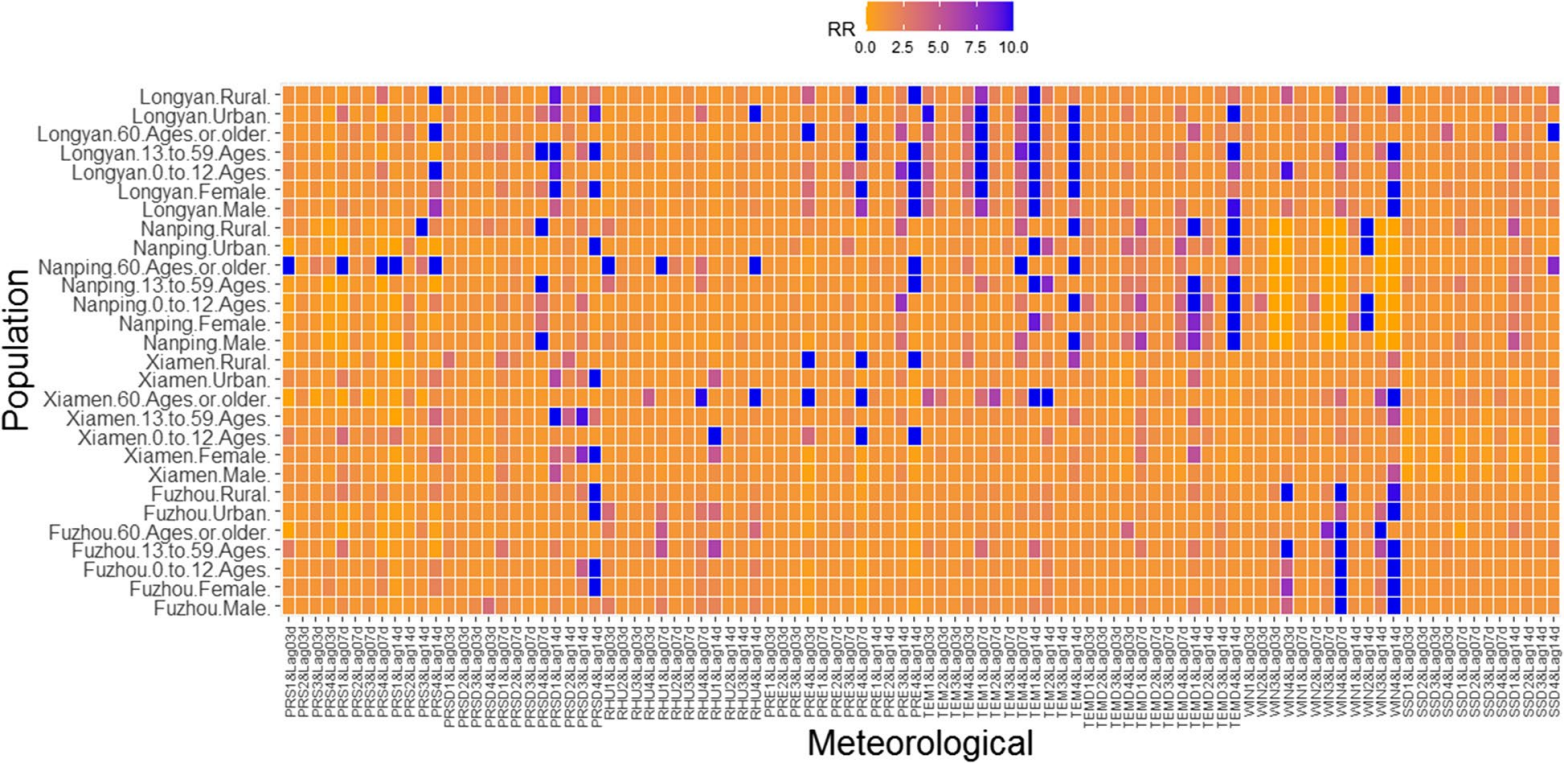
Cumulative effects of MFs on the risk of influenza in each population. (Note: (a). When RR > 10, it was counted as 10. (b). Meteorological values were divided into four grades, and the details are presented in Table 1. (c). The number of lag days was divided into three grades: 3 d, 7 d and 14 d.)

Additional characteristics of the impact of MFs on influenza in each population are presented in Fig. 5.

### Association between the Interaction of Different MFs with Influenza

**Fig. 6:**
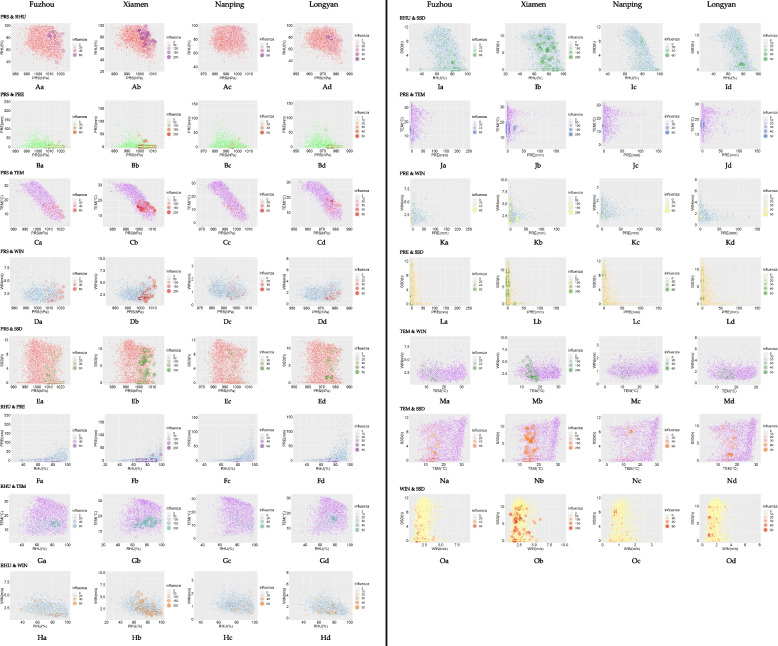
Associations between the interaction of different MFs and influenza

Among the four cities, the interaction between different MFs and influenza was the most obvious in Xiamen and the least obvious in Nanping. Overall, when PRS was between 1005–1015 hPa, RHU > 60%, PRE was low, TEM was between 10–20 °C, and WIN was low, the interaction between different MFs and influenza was most obvious. However, the value range was not completely consistent among the four cities. For instance, the PRS range of Fuzhou was 1005–1025 hPa, Xiamen was 1005–1015 hPa, and Longyan was 972–985 hPa. In addition, the interaction between SSD and other MFs was not obvious in the impact on influenza. Additional characteristics of the association between the interactions of different MFs with influenza are presented in Fig. 6.

### LSTM Forecast

**Fig. 7:**
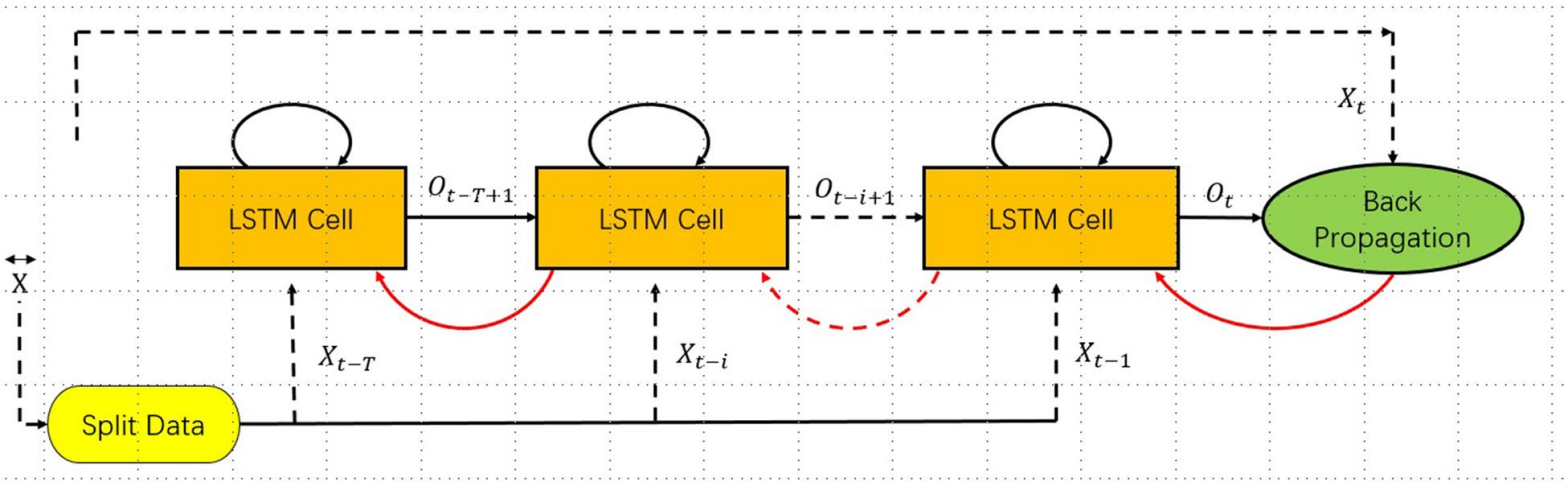
A brief analysis of the LSTM operation process in this study

In this study, we designed a prediction algorithm based on LSTM to capture the temporal relationship in the sequence. The network model was trained with historical data until it converged. The historical time series data included time, climate data and influenza incidence data. After coding, LSTM was input to capture the timing relationship, and then the fully connected layer was entered after coding and splicing to output the timing prediction. A brief description of the operation is shown in Fig. 7.

First, the meteorological and influenza data from 2010 to 2018 were trained and modelled to predict the daily cases of influenza in 2019. Then, the data from 2010 to 2019 were trained and modelled to predict the daily cases of influenza in 2020. Finally, the data from 2010 to 2020 were trained and modelled to predict the daily cases of influenza in 2021.

**Fig. 8:**
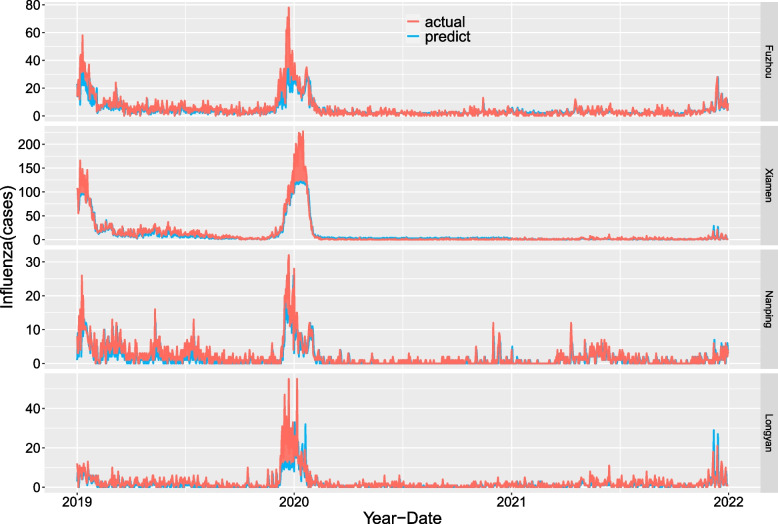
Influenza forecasts and true values for the four cities from 2019 to 2021 based on LSTM

The LSTM forecast evaluation indicators of the four cities are shown in Table 3. The RMSE, MAE, MAPE, and SMAPE values of the forecast evaluation indicators for 2019, 2020 and 2021 decreased yearly. The true and forecast values for 2019–2021 are shown in Fig. 8. The results show that the predicted and actual values matched well and that the accuracy was high.

**Table 3:** LSTM prediction evaluation indicators for the four cities

**Training years**	**Prediction year**	**Evaluation indicator**	**Fuzhou**	**Xiamen**	**Nanping**	**Longyan**
2010 ~ 2018	2019	RMSE	8.21	11.57	3.88	5.27
		MAE	4.65	7.19	2.49	2.54
		MAPE	0.52	0.47	0.76	0.88
		SMAPE	0.54	0.66	1.12	1.52
2010 ~ 2019	2020	RMSE	2.89	16.78	2.06	2.95
		MAE	1.89	6.50	0.88	1.35
		MAPE	0.59	0.66	0.75	0.79
		SMAPE	0.79	1.31	1.44	1.48
2010 ~ 2020	2021	RMSE	2.40	3.12	1.63	2.84
		MAE	1.75	2.43	1.00	1.47
		MAPE	0.55	0.84	0.69	0.73
		SMAPE	0.84	1.03	1.44	1.22

## Discussion

MFs can affect the onset of influenza by influencing the reproduction, growth and spread of pathogens, as well as human behaviour, immunity, etc.

This study showed that 8 meteorological indicators had different degrees of influence on influenza in four cities in Fujian Province.

This study found that both low and high PRS were risk factors for influenza in the four cities. Figure 2 shows that the association between an influenza season epidemic and high PRS (cold high PRS), as well as the subepidemic season and low PRS, was relatively clear, consistent with the results of DLNM analysis. Low, medium and high PRS were risk factors for women, children aged 0 to 12 years and rural populations with influenza, possibly because these populations with extreme cold weather and high PRS were more susceptible to influenza infection. It may be that women and children aged 0 to 12 years have lower immunity, and their risk of viral infection is greater. Due to the urban heat island effect, the TEM in rural areas is lower, so they are more susceptible to cold high PRS.

TEM is the MF that has the greatest impact on influenza, primarily due to the impact of low TEM [16, 33, 34]. However, the results of this study showed that low (< 9 °C) and high (> 23 °C) TEMs were risk factors for influenza in the four cities. Low TEM is mainly distributed during the winter and spring influenza epidemic seasons. Low TEM can reduce the body's resistance, and this is during cold high PRS. Cold high PRS often brings dryness, the nasal mucosa is prone to fine cracks, and viruses can easily invade. Studies have also reported that low TEM enhanced the viability of influenza viruses and provided more appropriate conditions for transmission [16, 35]. In low TEM weather, people are more active indoors, and there is less ventilation through windows and doors, which is conducive to the spread of the virus. High TEMs are distributed in the summer and autumn of the small peak period in influenza, when the highest TEM has often been reached. During this time, home and public places generally use air conditioning, indoor low-temperature drying is conducive to virus growth, the indoor and outdoor temperature difference is larger, autoimmunity is reduced, and the long-term sealed indoor environment also easily promotes the spread of influenza, which may explain why high TEM is a risk factor for influenza onset. At the same time, this is also one of the important reasons for the impact on influenza caused by high TEM during times of low PRS.

The effect of PRSD, TEMD and WIN on influenza has rarely been reported. However, this study showed that these three indicators are important for studying the effects of MFs on influenza. DLNM analysis showed that rural people are more sensitive to changes in PRS. The effect of high TEMD was more significant on influenza, while Fig. 2 shows that TEMD had two peak periods per year, which were distributed between the low peak period and peak period of TEM. Figure 3 also shows that TEMD and TEM were significantly positively correlated, so a high TEMD contributed more to the secondary peak period of influenza in summer and autumn. This study also showed that the risk of influenza onset increased with WIN (> 6 m/s) (Nanping was > 2 m/s). Figure 2 shows that WIN had two peak periods per year, consistent with the TEMD distribution period, which may aggravate the somatosensory TEMD effect, especially in the winter and spring low temperature seasons, which increases the risk of influenza virus infection by reducing the body's immunity. Therefore, PRSD, TEMD and WIN are important indicators to explore the impact of MFs on influenza.

This study showed that the interaction between different MFs and influenza was most obvious when PRS was between 1005–1015 hPa, RHU was greater than 60%, PRE and WIN were low, and TEM was between 10–20 °C. Because these MFs were superimposed in late winter and early spring in southern China, when cold high PRS and RHU were high but PRE and WIN were not high, this suggests that in the winter and spring influenza epidemic seasons, the interaction between different MFs and influenza was significant. However, different research reports were not completely consistent. For instance, this study showed that the interaction effect between higher humidity and higher air pressure and lower air temperature on influenza was obvious. The peaks of influenza infection risk were observed at low temperatures or at high temperatures with high relative humidity, although there were differences between Shanghai, Hong Kong, and British Columbia [36]. The interaction effect of low relative humidity with low ambient temperature and high air pressure aggravated the incidence of influenza in Lanzhou [12].

However, the effects of MFs on influenza were also inconsistent in these four cities. One reason was that the range and peak value of MFs that affected influenza were inconsistent in the four cities. For instance, the peak of low PRS impact in Fuzhou and Xiamen was concentrated in 975 ~ 979 hPa, high PRS referred to > 112 hPa, while in Longyan, low PRS impact peak appeared at 964 hPa, and high PRS was only 990 hPa. Another reason was that the influence mode of MFs on influenza was not completely consistent. For instance, the TEM in Fuzhou and Xiamen was also a risk factor for influenza onset at 10 to 15 °C, showing a wavy type between 3 and 33 °C, while in Nanping and Longyan, it showed a "U" type between -2 and 32 °C. Low RHU had a significant effect on influenza in Fuzhou, Xiamen and Nanping, while high RHU had a more significant effect in Longyan. Furthermore, there were completely different patterns of association between MFs and influenza in the different cities. For instance, the increase in PRE in Xiamen and Longyan increased the risk of influenza, while the opposite effect was observed in Fuzhou and Nanping. TEMD seemed to have the opposite effect in Xiamen and Nanping. The interaction between PRS and other MFs (except SSD) was obvious in the impact on influenza, but the values in the four cities were not completely consistent; the PRS range of Fuzhou was 1005–1025 hPa, the range in Xiamen was 1005–1015 hPa, and the range in Longyan was 972–985 hPa.

Many factors may affect the relationship between MFs and influenza.

Supplemental Fig. 1 and Supplemental Table 1 show that Fuzhou and Xiamen are coastal cities, and their meteorological conditions are relatively consistent. Therefore, their MFs have many similarities in the impact on influenza. However, Xiamen has a small area, and the meteorological stations are more representative, while Fuzhou has a vast area, and there is a large difference between urban and rural MFs and the geographical environment. Therefore, there are some inconsistencies between Fuzhou and Xiamen regarding the impact of MFs on influenza. Supplemental Fig. 1 and Supplemental Table 1 also show that Nanping and Longyan have meteorological characteristics of small WIN and SSD and large TEMD. In addition, Nanping also has a low TEM and a high RHU, while the daily minimum, daily maximum and daily average of PRS in Longyan are very low. These meteorological and geographical differences undoubtedly affect the meteorological analysis of influenza risk to a large extent.

In 2021, the urbanization rate of Xiamen's resident population reached nearly 90%. Moreover, Xiamen is economically developed and a tourist city. It has a large floating population and a large population density. These are also important reasons for the high number of influenza cases. The medical and health conditions are superior in Xiamen, and the influenza case report rate is high. Therefore, the reported incidence (563.83/100,000 people) rate of influenza in Xiamen is the highest among the four cities and the highest in Fujian Province. However, Nanping and Longyan account for a high proportion of rural areas, and the urbanization rate is less than 60%. The density of the resident population is low, with fewer aggregation activities. Moreover, they are more mountainous areas, which makes the vertical and horizontal distribution of the population more dispersed. The phenomenon of rural children and elderly individuals staying behind is very common in China, and they are susceptible groups, so they are vulnerable to influenza. However, most rural people with ILI seek medical treatment in village clinics, and case information from village clinics is more often than not required to be reported to the China Disease Prevention and Control Information System, so the reported number of influenza cases underestimates the true value. In addition, during the holidays, especially the Spring Festival, which is during the season of high incidence of seasonal influenza, the population flows frequently, which is conducive to the spread of influenza. Xiamen and Fuzhou are obvious cities of population import. There is the phenomenon of "empty Spring Festival city" in China due to the siphon effect of large cities. At the same time, the return of migrant workers and students has changed the phenomenon of children and elderly individuals staying behind. The impact of these factors on different cities may be inconsistent, but there is no detailed research report at present, so the impact on the relationship between MFs and influenza needs further study.

Moreover, influenza vaccination is the most effective means to prevent influenza. In Europe, influenza vaccination rates on average are 45% [37]. In the 2017–2018 influenza season, 57.9% of children between the ages 6 months and 17 years were vaccinated in the United States [38, 39]. But in mainland China, the influenza vaccination rate was extremely low, had reached the highest point in 2010 at 37.3% but dropped to 7% in 2017 [40]. The vaccination rate needs to be at least 50%-60% for herd immunity to occur. Therefore, the difference in the influenza vaccination rate among the four cities is very slight.

The dynamic assessment and prediction of epidemics is an important part of the prevention and control of infectious diseases. In terms of prediction, the methods commonly used in the prediction autoregressive integrated moving average (ARIMA) model, susceptible-infectious-recovered (SIR) model, and recurrent neural networks (RNNs) have good performance, but the prediction accuracy is not high. The ARIMA model is not suitable for modelling nonlinear relationships, the SIR model cannot add MFs and cannot make full use of the information in the multidimensional input data, gradient extinction easily occurs in RNNs, and the problem of long-distance dependence cannot be handled [31, 41–44].

With the development of artificial intelligence, machine learning algorithms have demonstrated advantages in prediction and recognition [45–48]. LSTM is an advanced RNN with the ability to learn time patterns and store useful memories longer [48]. Due to its unique design structure, LSTM has the ability to solve gradient extinction problems, handle nonlinear relationships, and incorporate MFs. It is also suitable for processing and predicting important events with very long intervals and delays in time series. Therefore, this study used 8 meteorological indicators and historical influenza data to construct an LSTM model, which was actually a multivariate LSTM model prediction that was very suitable for predicting the daily cases of influenza.

The results of this study showed that the RMSE, MAE, MAPE, and SMAPE of the 2019–2021 yearly predictions and evaluation of influenza cases in the four cities were very low by using a multivariate LSTM model of MFs. Previous studies have reported that LSTM is superior to other methods in predicting viral infectious disease [20, 21, 49]. Different studies may have different data orders of magnitude, so the RMSE and MAE are not suitable for comparison with other reports. However, the MAPE and SMAPE are not affected by this. This study was consistent with other similar studies with low values [20, 21, 49]. Figure 8 also confirms that the true and forecast values of the four cities are highly consistent. This shows that the eight meteorological indicators used in this study can accurately predict the daily cases of influenza through LSTM models.

It is worth mentioning that, first, there are regional differences in the impact of meteorology on influenza, so it is necessary to evaluate and predict by city. Second, at present, extreme weather events occur frequently, and the effect of climate change is obvious. If the prediction time is too long, the prediction value may be unstable and inaccurate in the later stage, which will lose practical usefulness. In addition, environmental factors, people's living habits, government policies and other factors will also have a certain impact on the occurrence and development of influenza, and historical cases of influenza also participate in LSTM training modelling. Therefore, it is recommended to adopt a forecast cycle of half a year or one year, incorporate newly collected meteorological and influenza data in a timely manner, revise the model, and reforecast. The third is to avoid overfitting. LSTM models risk overfitting or underfitting, which often results in poor prediction performance [21, 50]. When the number of memory neurons is less than 32 or the number of training rounds is less than 250, the performance of the model deteriorates [21].

This study also has some limitations. First, the influenza data of the study came from the China Disease Prevention and Control Information System, which is reported by medical and health institutions at all levels in various places. Some patients who have cold symptoms only go to pharmacies to buy medicines for treatment or go to village clinics for treatment without receiving case reports. Therefore, the number of reported influenza cases may underestimate reality. The proportion of these conditions in various cities may not be consistent, which may affect the relationship between meteorology and influenza. Second, in the context of the COVID-19 pandemic, the widespread implementation of nonpharmacological interventions (e.g., global travel, mask use, physical distancing, and stay-at-home orders) has reduced the spread of some viral respiratory pathogens [51–53], particularly in cases where schools prone to cluster influenza outbreaks have also been suspended due to the COVID-19 pandemic. And School-age children have been described as the driving force behind the family and community epidemics during the influenza season [53–55]. As a result, the number of reported influenza cases has dropped considerably, so the influenza reporting data of the past two years may have some impact on the research and prediction accuracy of the impact of meteorology on the risk of influenza in 2010–2021. Third, a stratified analysis of influenza aetiology has not been carried out because only a portion of the cases have undergone laboratory pathogen testing, often in medical institutions for influenza detection. If only this subset of the cases is selected, it will limit the usefulness of the study results.

## Conclusion

Larger meteorological differences between regions can lead to significant regional differences in its impact on the risk of influenza. All eight MFs studied had an impact on influenza in four cities, among them the impact of WIN, TEM, PRS, PRSD and SSD was obvious and relatively consistent in the four cities, however, the impact of RHU and PRE on influenza varied greatly in the 4 cities. The impact of both low and high values of MFS studied on the risk of influenza was more significant, especially for women, 0 ~ 12-year-old, ≥ 60-year-old and rural population. The LSTM model, combined with these eight MFs, was highly accurate in predicting the daily cases of influenza in 4 cities. These MFs and prediction models can be incorporated into the influenza early warning and prediction system of each city to make preparations to help relevant departments deal with the possible outbreak of influenza and can be used as a reference for each city to formulate prevention strategies and risk prediction for adjusting people's lifestyles.

## Supplementary Information

**Additional file 1. **

## Data Availability

The datasets that support the findings of this study are available from Fujian Provincial Centre of Disease Control and Prevention, Fujian Climate Center and meteorological data network of the China Meteorological Administration, but restrictions apply to the availability of these data, which were used under license for the current study, and so are not publicly available. Data are however available from the authors upon reasonable request and with permission of these three institutions (E-mail: hszhu33@126.com).

## Abbreviations

AIC: Akaike information criterion
ARIMA: Autoregressive integrated moving average
CDC: The US Centers for Disease Control and Prevention
CI: Confidence interval
COVID-19: Corona virus disease 2019
DLNM: Distribution lag nonlinear models
GBD: Global Burden of Disease Study
ILI: Influenza-like illness
LSTM: Long short-term memory
MAE: Mean absolute error
MAPE: Mean absolute percentage error
MF: Meteorological factor
PRE: Precipitation
PRS: Pressure
PRSD: Pressure difference
RHU: Relative humidity
RMSE: Root mean squared error
RNNs: Recurrent neural networks
RR: Risk ratio
SIR: Susceptible-infectious-recovered
SMAPE: Symmetric mean absolute percentage error
SSD: Sunshine duration
TEM: Temperature
TEMD: Temperature difference
WHO: World Health Organization
WIN: Wind speed
