# Correction: Study on the influence of meteorological factors on influenza in different regions and predictions based on an LSTM algorithm

**DOI:** 10.1186/s12889-023-15164-2

**Published:** 2023-02-07

**Authors:** Hansong Zhu, Si Chen, Wen Lu, Kaizhi Chen, Yulin Feng, Zhonghang Xie, Zhifang Zhang, Lingfang Li, Jianming Ou, Guangmin Chen

**Affiliations:** 1 Emergency Response and Epidemic Management Institute, Fujian Center for Disease Control and Prevention, Fuzhou, 350012 Fujian China; 2 Fujian Provincial Key Laboratory of Zoonosis Research, Fuzhou, 350012 Fujian China; 3 The practice base on the school of public health Fujian Medical University, Fuzhou, 350012 Fujian China; 4 Climate Assessment Office of Fujian Climate Center, Fuzhou, 350007 Fujian China; 5 Shengli Clinical Medical College of Fujian Medical University, Department of Health Management of Fujian Provincial Hospital, Fuzhou, 350001 Fujian China; 6 College of Computer and Data Science, Fuzhou University, Fuzhou, 350108 Fujian China; 7 School of Public Health, Fujian Medical University, Fuzhou, 350108 Fujian China; 8 Science and Technology Information and Management, Fujian Center for Disease Control and Prevention, Fuzhou, 350012 Fujian China

**Correction: BMC Public Health 22, 2335 (2022)**

**https://doi.org/10.1186/s12889-022-14299-y**

The original publication of this article [1] contained an incorrect funding number. The incorrect and correct funding information is shown in this correction article. The original article has been updated.

Incorrect: 2020J0111

Correct: 2020J01094
