# Supplementary material for: Study on the influence of meteorological factors on influenza in different regions and predictions based on an LSTM algorithm
Source: BMC Public Health. 2022 Dec 13;22:2335. doi: 10.1186/s12889-022-14299-y (PMC9745690; doi:10.1186/s12889-022-14299-y)
Supplement: Supplementary file 1 — Additional file 1. [file 12889_2022_14299_MOESM1_ESM.docx]

**Appendix**

**Supplemental Figure**：

**
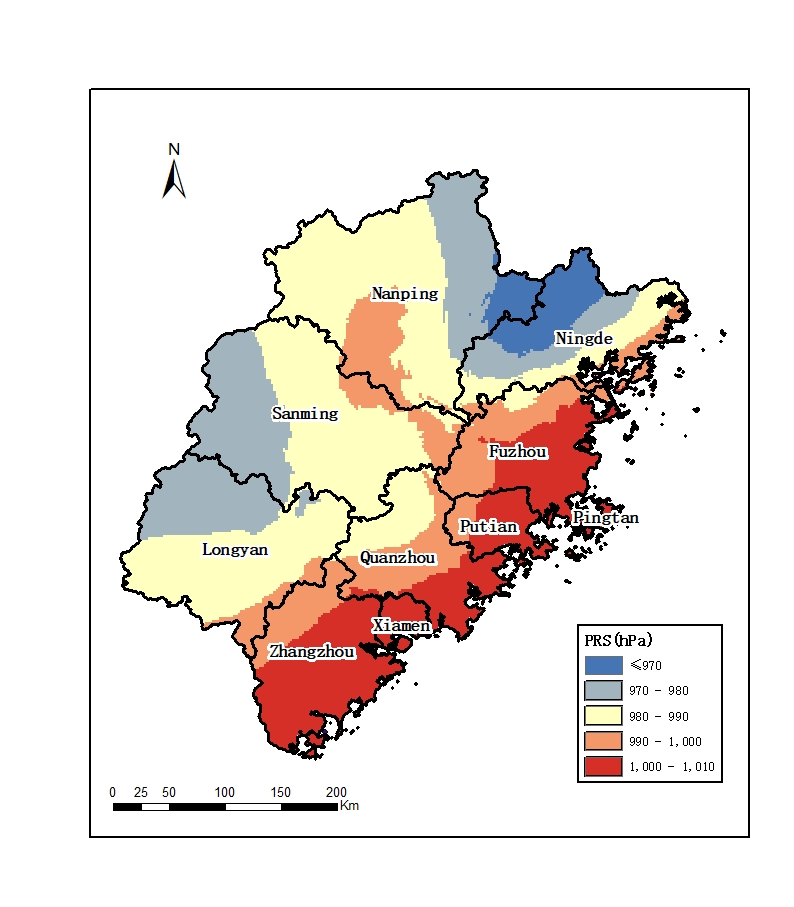

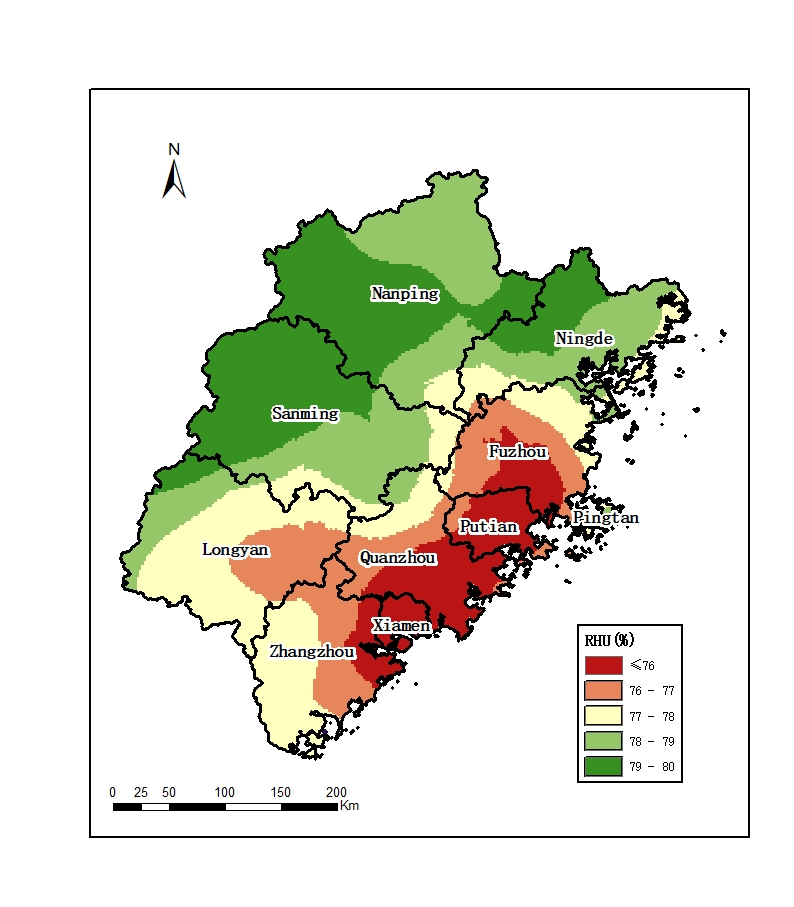

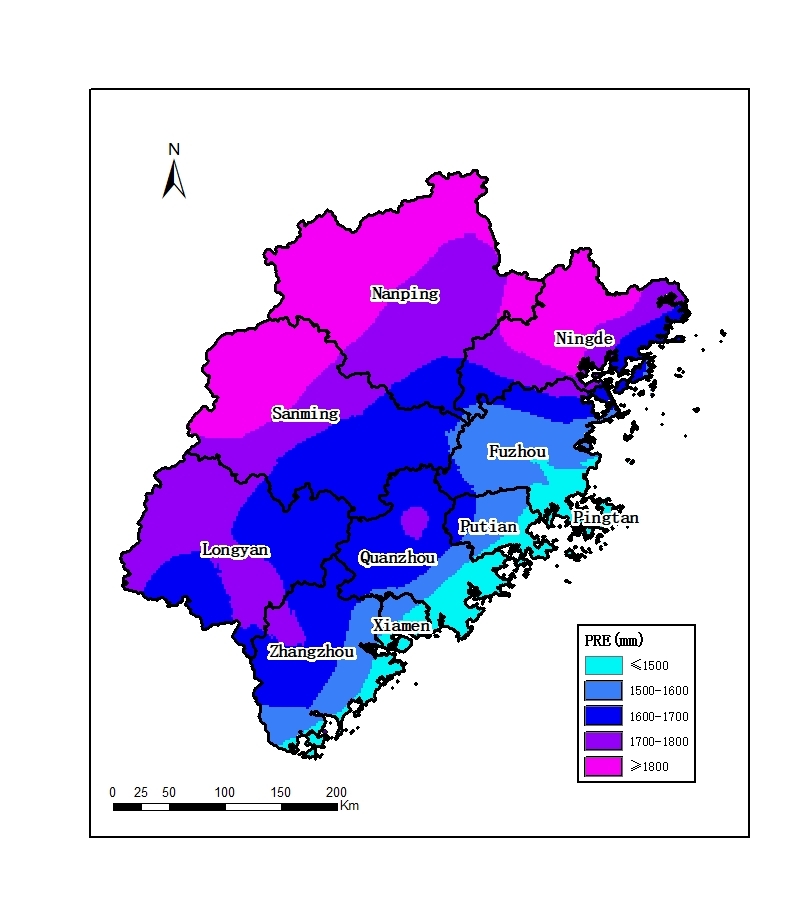
**

PRS RHU PRE

**
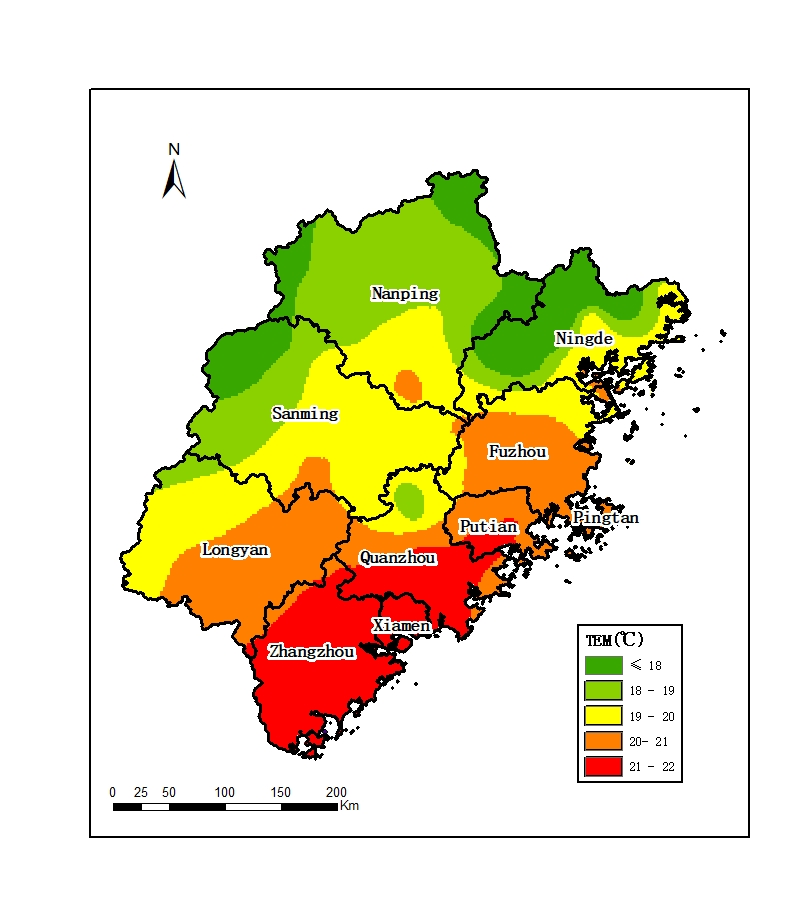

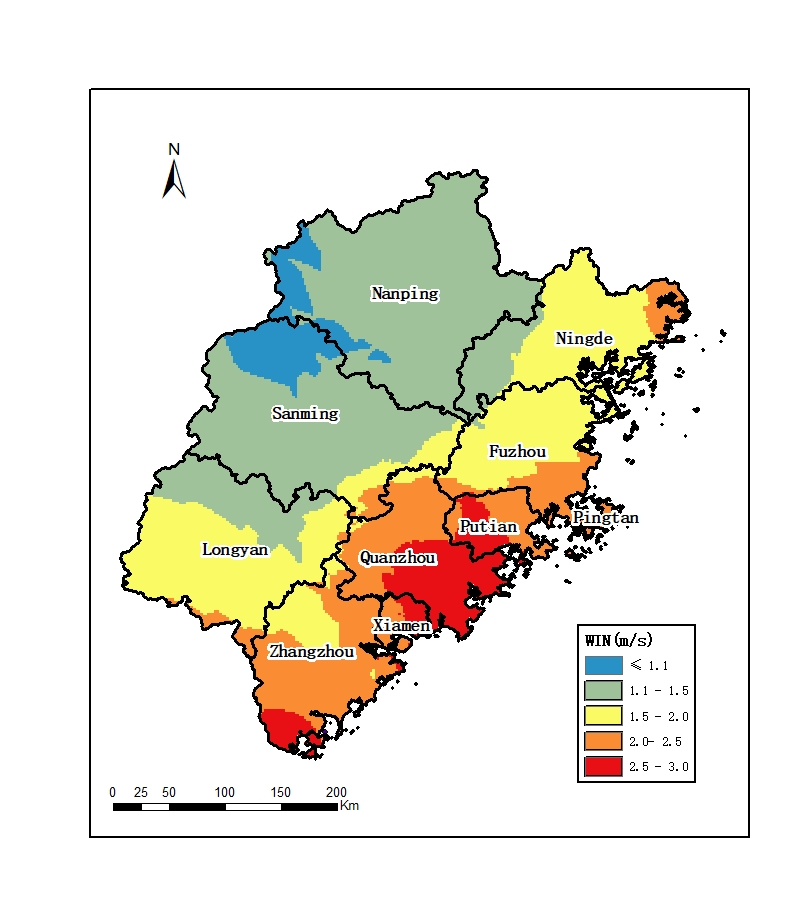

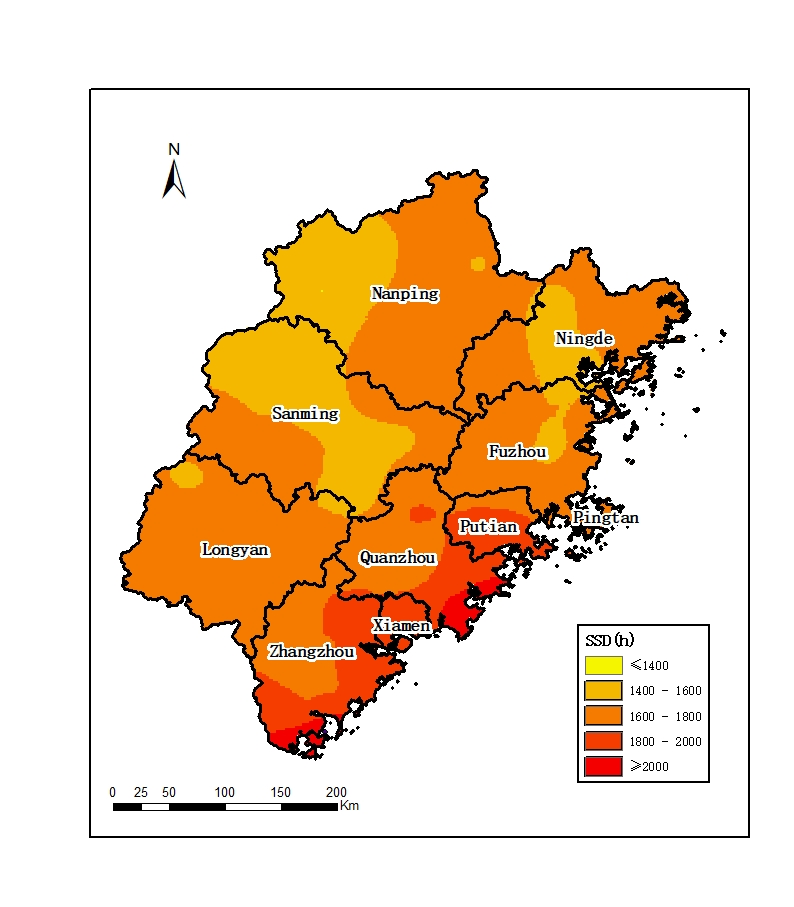
**

TEM WIN SSD

**Supplemental Figure 1** Characteristics distribution of MFs in Fujian Province


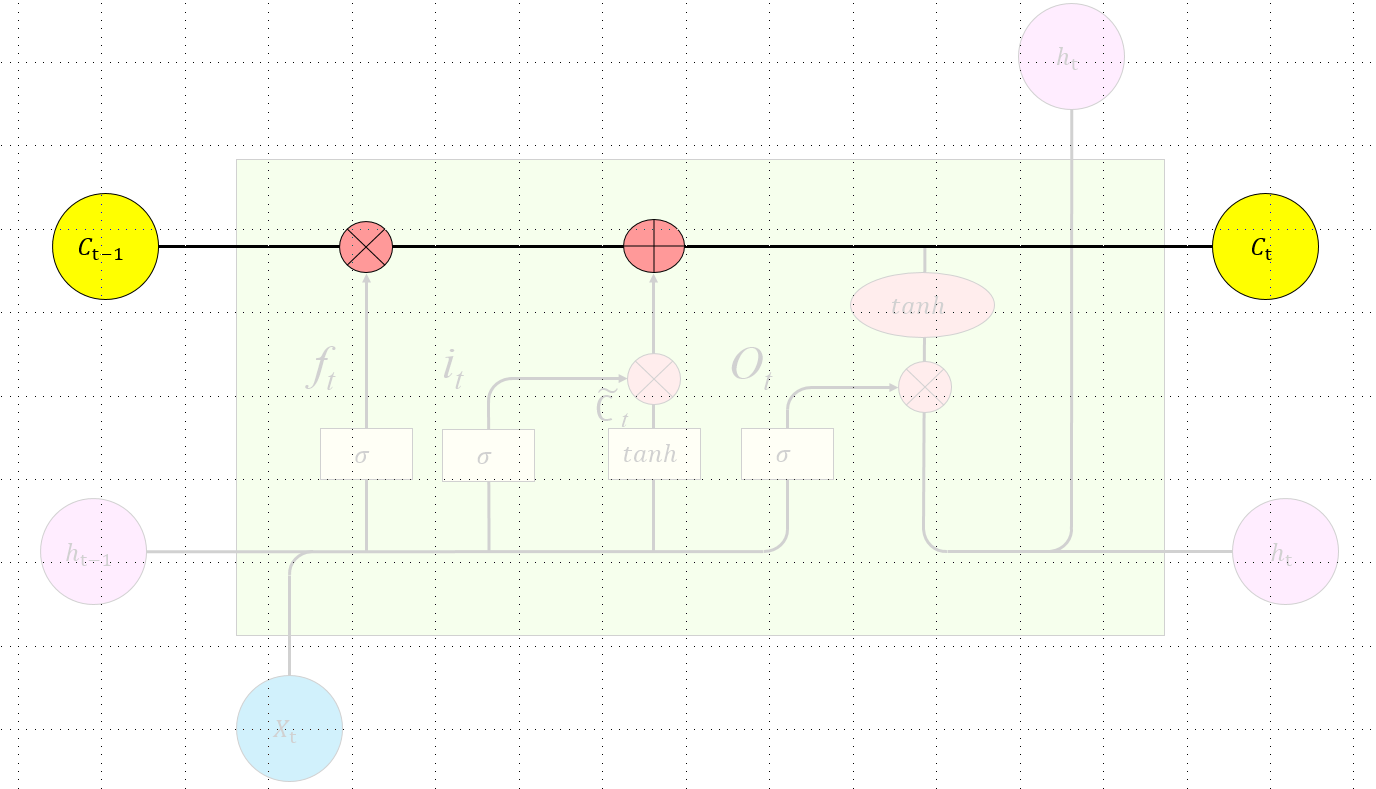


**Supplemental Figure 2** LSTM core idea structure diagram

**Supplemental Attachment 1** Basic principle of LSTM

The neurons change their state information with the previous data flow, process the current input data according to the current state and output the results. This structure gives neurons a certain memory ability. LSTM has a well-designed structure called a gate to remove or add information to the neuron state to avoid the problem of long-term dependence and retain the long-term information in the sequence. Gates provide a means for information to be passed selectively. LSTM has three gates, a forgetting gate, an input gate and an output gate, to protect and control the state of neurons.

The core idea of LSTM is shown in Supplemental Figure 2.

The first step is to decide what information to discard from the neuron state, which is done through the sigmoid layer of the "forgetting gate". *h*_t−1_ represents the output of the previous neuron state, *X*_t_ represents the input of the current neuron state, and *σ* represents the sigmoid function. The sigmoid layer outputs a numeric value between 0 and 1, denoting how much of each part can pass through, with 0 representing complete discard, and 1 representing complete retention. The expression is as follows:

*f_t_* = *σ*(*W_f_* [*h_t_*_-1_, *x_t_* ] + *b_f_*) (1)

The second step is to determine what kind of new information is stored in the neuron state. There are two parts here: first, the sigmoid layer of the "input gate" determines which value will be updated; then, the tanh layer creates a new candidate value vector *_t_* (a value between -1 and 1) that is added to the state and multiplied by the value of the sigmoid function, updating the old neuron state, *C_t_*_-1_ is updated to *C_t_*, and finally the output determines the part to output. The expression is as follows:

*i_t_* = *σ*(*W_i_* $\cdot$ [*h_t_*_-1_, *x_t_* ] + *b_i_*) (2)

$\tilde{C}$*_t_* = tanh(*W_c_* $\cdot$ [*h_t_*_-1_, *x_t_* ] + *b_c_* (3)

*C_t_* = *f_t_* $\cdot$ *C_t-1_* + *i_t_* $\cdot$ $\tilde{C}$*_t_* (4)

Finally, the output must be determined by the "output gate". First the sigmoid layer is run to determine which part of the neuron state to output; then, the neuron state is processed by tanh (given a value between -1 and 1) and multiplied by the output of the sigmoid gate, and finally, the determined part is output. The expression is as follows:

*o_t_* = *σ*(*W_o_* $\cdot$ [*h_t_*_-1_, *x_t_* ] + *b_o_*) (5)

*h_t_* = *o_t_* $\cdot$ tanh(*C_t_*) (6)

**Supplemental Table 1** Descriptive statistics of the variables for daily influenza cases and MFs in the four cities

| **Variables** | **Fuzhou** | | | |  | **Xiamen** | | | |  | **Nanping** | | | |  | **Longyan** | | | |  | **P** |
| --- | --- | --- | --- | --- | --- | --- | --- | --- | --- | --- | --- | --- | --- | --- | --- | --- | --- | --- | --- | --- | --- |
|  | **Min** | **M50** | **Max** | **Mean±SD** |  | **Min** | **M50** | **Max** | **Mean±SD** |  | **Min** | **M50** | **Max** | **Mean±SD** |  | **Min** | **M50** | **Max** | **Mean±SD** |  |  |
| Influenza (cases) | 0.00 | 2.00 | 78.00 | 3.13±5.29 |  | 0.00 | 0.00 | 227.00 | 4.87±18.05 |  | 0.00 | 0.00 | 67.00 | 0.98±2.85 |  | 0.00 | 0.00 | 56.00 | 1.30±3.07 |  | 0.00 |
| PRS (hPa) | 978.50 | 1005.00 | 1026.20 | 1005.00±7.49 |  | 974.50 | 997.90 | 1017.20 | 997.80±6.53 |  | 967.60 | 991.70 | 1015.40 | 991.80±7.45 |  | 949.90 | 970.70 | 990.20 | 970.70±5.94 |  | 0.00 |
| PRSD (hPa) | 1.40 | 4.50 | 24.50 | 4.80±1.75 |  | 1.70 | 4.10 | 39.70 | 4.29±1.41 |  | 1.60 | 4.60 | 15.90 | 4.82±1.47 |  | 1.60 | 4.40 | 12.40 | 4.53±1.22 |  | 0.00 |
| RHU (%) | 27.00 | 73.00 | 100.00 | 73.45±12.53 |  | 23.00 | 77.00 | 100.00 | 75.63±12.95 |  | 36.00 | 80.00 | 100.00 | 79.99±10.16 |  | 25.00 | 74.50 | 100.00 | 74.26±10.44 |  | 0.00 |
| PRE (mm) | 0.00 | 0.00 | 244.40 | 4.22±12.37 |  | 0.00 | 0.00 | 172.70 | 3.38±11.54 |  | 0.00 | 0.00 | 154.20 | 5.06±12.49 |  | 0.00 | 0.00 | 152.30 | 5.07±13.40 |  | 0.00 |
| TEM (°C) | 2.30 | 21.40 | 33.40 | 20.83±7.02 |  | 3.90 | 22.30 | 32.80 | 21.55±6.05 |  | -1.60 | 20.00 | 31.80 | 19.04±7.73 |  | 1.90 | 21.80 | 31.00 | 20.76±6.16 |  | 0.00 |
| TEMD (°C) | 0.60 | 7.50 | 17.70 | 7.41±3.25 |  | 0.80 | 6.70 | 15.00 | 6.70±2.26 |  | 0.70 | 9.20 | 21.20 | 9.08±4.12 |  | 1.10 | 9.30 | 22.30 | 9.24±3.92 |  | 0.00 |
| WIN (m/s) | 0.55 | 2.10 | 9.10 | 2.20±0.74 |  | 0.60 | 2.50 | 9.80 | 2.63±0.97 |  | 0.10 | 1.00 | 3.50 | 1.08±0.37 |  | 0.00 | 1.60 | 7.80 | 1.73±0.71 |  | 0.00 |
| SSD (h) | 0.00 | 3.40 | 12.40 | 4.10±3.90 |  | 0.00 | 5.90 | 13.00 | 5.35±4.07 |  | 0.00 | 4.00 | 12.40 | 4.28±3.88 |  | 0.00 | 5.00 | 12.80 | 4.91±3.92 |  | 0.00 |
